# Diet-Induced Obesity Exacerbates Auditory Degeneration via Hypoxia, Inflammation, and Apoptosis Signaling Pathways in CD/1 Mice

**DOI:** 10.1371/journal.pone.0060730

**Published:** 2013-04-26

**Authors:** Juen-Haur Hwang, Chuan-Jen Hsu, Wei-Hsuan Yu, Tien-Chen Liu, Wei-Shiung Yang

**Affiliations:** 1 Graduate Institute of Clinical Medicine, College of Medicine, National Taiwan University, Taipei, Taiwan; 2 Department of Otolaryngology, Buddhist Dalin Tzu-Chi General Hospital, Chiayi, Taiwan; 3 School of Medicine, Tzu Chi University, Hualien, Taiwan; 4 Department of Otolaryngology, National Taiwan University, Hospital, Taipei, Taiwan; 5 Institute of Biochemistry and Molecular Biology, College of Medicine, National Taiwan University, Taipei, Taiwan; 6 Department of Internal Medicine, National Taiwan University, Hospital, Taipei, Taiwan; 7 Research Center for Developmental Biology and Regenerative Medicine, National Taiwan University, Taipei, Taiwan; Universidad Pablo de Olavide, Centro Andaluz de Biología del Desarrollo-CSIC, Spain

## Abstract

The aim of this study was to investigate the mechanisms of diet-induced obesity on hearing degeneration in CD/1 mice. Sixty 4-week-old male CD/1 mice were randomly and equally divided into 2 groups. For 16 weeks, the diet-induced obesity (DIO) group was fed a high fat diet and the control group was fed a standard diet of 13.43 % kcal fat. The morphometry, biochemistry, auditory brainstem response thresholds, omental fat, and histopathology of the cochlea were compared between the beginning and end of the study (4 vs. 20 weeks old). The results show that the body weight, fasting plasma triglyceride concentrations, and omental fat weight were higher in the DIO group than in the control group at the end of experiment. The auditory brainstem response thresholds at high frequencies were significantly elevated in the DIO group compared to those of the control group. Histology studies showed that, compared to the control group, the DIO group had blood vessels with smaller diameters and thicker walls in the stria vascularis at the middle and basal turns of the cochlea. The cell densities in the spiral ganglion and spiral ligament at the basal turn of the cochlea were significantly lower in the DIO group. Immunohistochemical staining showed that hypoxia-induced factor 1 (HIF-1), tumor necrosis factor alpha (TNF-α), nuclear factor kappa B (NF-κB), caspase 3, poly(ADP-ribose) polymerase-1, and apoptosis inducing factor were all significantly more dense in the spiral ganglion and spiral ligament at the basal turn of cochlea in the DIO group. Our results suggest that diet-induced obesity exacerbates hearing degeneration via increased hypoxia, inflammatory responses, and cell loss in the spiral ganglion and spiral ligament and is associated with the activation of both caspase-dependent and -independent apoptosis signaling pathways in CD/1 mice.

## Introduction

Obesity-related cardiometabolic disorders are known to be associated with hearing impairment [1]-[5]. Whether obesity per se could lead to hearing impairment was uncertain until the recent publication of 2 independent studies [6], [7]. In a European population-based multicenter study, occupational noise, smoking, and a high body mass index (BMI) were found to be risk factors for hearing impairment [6]. Our own epidemiological study also showed that waist circumference (WC), a measurement of visceral or central obesity, is an independent risk factor for hearing impairment in Taiwanese [7]. However, the detailed mechanisms of obesity-related hearing impairment have seldom been explored. Obesity has been hypothesized to directly impair the function of many organ systems via obesity-related oxidative stress and lipotoxicity [8]–[10]. Obesity-induced inflammation also results in the infiltration of macrophages and the release of proinflammatory cytokines, which could exacerbate end-organ damage and cell apoptosis in the heart, kidney, pancreas, liver, and skeletal muscle via the caspase-dependent signaling pathway [10]. Lipotoxicity can also lead to cell death via caspase-independent signaling pathways, as demonstrated in pancreatic beta-cell lines [11]. However, no reports have addressed the effects of lipotoxicity and its related apoptosis signaling pathways on the peripheral auditory organ.

In this study, we aimed to address the possible mechanisms of diet-induced obesity (DIO) on hearing impairment in CD/1 mice. The CD/1 mouse strain is particularly well-suited to this type of study because these mice have early onset of hearing impairment, beginning at 4 weeks of age. This feature allowed us to observe the effects of DIO on hearing degeneration earlier in the lifespan of the mice, thus avoiding biochemical insults other than obesity itself [12]. In the cochlea of CD/1 mice, a prominent decline in spiral ganglion (SG) neuron density occurs at a young age. By the age of 6 months, these mice have a severe loss of SG neurons and fibrocytes in spiral ligaments (SL) and the Organ of Corti in the cochlear basal turn [12]–[14]. The mechanisms of degeneration in the SG may involve hypoxia, oxidative stress damage, increased expression of TNF-α, loss of neuronal protection, and activation of the caspase-dependent cell apoptosis signaling pathway [13]. No studies have investigated caspase-independent apoptosis signaling pathways in the auditory system of CD/1 mice.

We hypothesized that DIO could exacerbate hearing degeneration in CD/1 mice by increasing cochlear hypoxia and inflammation. In addition, a high fat diet might increase cell death in the inner ear through activation of both caspase-dependent and -independent apoptosis signaling pathways.

## Results

Table 1 shows that the baseline characteristics of the control and DIO groups were not significantly different at the beginning of the study. ABR thresholds were very similar between the 2 groups, as determined by click sounds using 8 kHz, 16 kHz, and 32 kHz tone bursts.

**Table 1:** Baseline characteristics of the both groups at the beginning of this study.

Mean±SD	Control group (n = 30)	DIO group (n = 30)	p values*
BW (gm)	28.1±2.4	27.6±3.6	0.6724
Plasma biochemistry			
TG (mg/dl)	83.7±9.3	87.5±11.6	0.6602
HDL-C (mg/dl)	117.1±25.1	122.9±24.4	0.7710
Glucose (mg/dl)	147.0±47.0	128.3±31.1	0.5493
ABR thresholds (dB SPL)			
Click sound	49.3±4.8	52.2±3.8	0.3109
8 kHz tone burst	37.0±8.5	36.3±8.2	0.9041
16 kHz tone burst	47.3±1.5	48.5±5.5	0.6728
32 kHz tone burst	55.2± 4.6	54.5± 3.8	0.7354

Abbreviations: SD: standard deviation; DIO: diet-induced obesity; BW: body weight; TG: triglyceride; HDL-C: high-density lipoprotein cholesterol; ABR: auditory brainstem response.

* Student's t-test.

Table 2 compares the control and DIO groups at the end of the study (20 weeks of age). BW (55.6±8.0 vs. 46.8±6.8 g; Student's t-test, p = 0.0002) and omental fat weight (2.6±0.7 vs. 1.4±1.1 g; Student's t-test, p = 0.0106) were significantly higher in the DIO group than in the control group. Fasting plasma TG was elevated significantly in the DIO group compared to the control group. Fasting HDL and glucose were not significantly different between the 2 groups. Both groups showed elevated ABR thresholds at the end of the study compared to those at the beginning at all frequencies tested. However, the ABR threshold was significantly higher in the DIO group than in the control group at 32 k Hz [98.7±1.0 vs. 92.5±4.6 dB sound pressure level (SPL); Student's t-test, p = 0.0080] and 16kHz sound stimulation (78.3±13.2 vs. 70.5±9.2 dB SPL; Student's t-test, p = 0.0240), and of borderline significance at 8 kHz (69.6±11.5 vs. 64.0±8.5 dB SPL; Student's t-test, p = 0.0637). However, the threshold was not significantly different between the 2 groups as determined by click sound stimulation, which reflects the thresholds around 4 kHz. These findings suggest that diet-induced obesity exacerbated hearing degeneration in CD/1 mice, especially at higher frequencies.

**Table 2:** Comparisons of the both groups at the end of this study.

Mean±SD	Control group (n = 30)	DIO group (n = 30)	p values*
BW (gm)	46.8±6.8	55.6±8.0	0.0002
Omentum fat (gm)	1.4±1.1	2.7±0.7	0.0106
Plasma biochemistry			
TG (mg/dl)	83.5±21.3	101.9±27.9	0.0380
HDL-C (mg/dl)	96.5±51.8	90.0±43.2	0.6314
Glucose (mg/dl)	199.0±36.5	197.0±70.0	0.9077
ABR thresholds (dB SPL)			
Click sound	66.6±8.3	70.9±11.9	0.1654
8 kHz tone burst	64.0±8.5	69.6±11.5	0.0637
16 kHz tone burst	70.5±9.2	78.3±13.2	0.0240
32 kHz tone burst	92.5±4.6	98.7±1.0	0.0080

Abbreviations: SD: standard deviation; DIO: diet-induced obesity; BW: body weight; TG: triglyceride; HDL-C: high-density lipoprotein cholesterol; ABR: auditory brainstem response.

* Student's t-test.

The mean thickness of the SV was not significantly different between the 2 groups at any of the 3 turns (Table 3). However, the mean internal diameter of vessels in the SV was significantly smaller in the DIO group than in the control group at the middle turn (3.9±0.3 vs. 4.8±0.5 µm; Student's t-test, p<0.0001) and the basal turn (3.7±0.5 vs. 4.5±0.5 µm; Student's t-test, p<0.0001). The ratio of the mean vessel wall thickness to its radius in the SV was significantly larger in the DIO group than in the control group at the middle turn (30.2±4.8 % vs. 27.3±5.0 %; Student's t-test, p = 0.0535) and basal turn (31.9±4.2 % vs. 28.3±2.9 %; Student's t-test, p = 0.0004).

**Table 3:** Observations of cochlear sections by H&E stain in both groups at the end of this study.

Mean±SD	Control	DIO	p value*
SV thickness ( µm)			
Apical turn	17.3±2.4	15.4±3.5	0.1015
Middle turn	17.8±2.1	17.7±2.9	0.8898
Basal turn	17.1±3.6	17.2±2.5	0.9519
Internal diameter of vessels in SV( µm)			
Apical turn	4.6±0.6	4.0±0.5	0.0008
Middle turn	4.8±0.5	3.9±0.3	<0.0001
Basal turn	4.5±0.5	3.7±0.5	<0.0001
Vessel wall to radius ratio in SV (%)			
Apical turn	29.6±1.6	30. 1±4.3	0.7683
Middle turn	27.3±5.0	30.2±4.8	0.0535
Basal turn	28.3±2.9	31.9±4.2	0.0004
Cell density of SG (/50 µm^2^)			
Apical turn	9.9±3.8	9.6±3.3	0.7691
Middle turn	12.8±3.8	11.6±3.0	0.0915
Basal turn	12.0±3.6	9.8±2.5	0.0092
Cell density of SL (/50 µm^2^)			
Apical turn	8.4±3.3	7.4±3.0	0.3110
Middle turn	9.8±3.2	8.2±3.0	0.2941
Basal turn	8.6±3.2	6.5±2.6	0.0515
IHC			
Apical turn	0.6±0.5	0.8±0.4	0.3953
Middle turn	0.6±0.4	0.8±0.3	0.1917
Basal turn	0.5±0.3	0.6±0.3	0.3202
OHCs			
Apical turn	2.0±1.2	2.1±0.8	0.8931
Middle turn	2.1±1.0	2.1±0.6	0.8125
Basal turn	1.2±0.7	0.8±0.8	0.1010

Abbreviations: SD: standard deviation; DIO: diet-induced obesity; SV: stria vascularis; SG: spiral ganglion; SL: spiral ligament; IHC: inner hair cell; OHCs: outer hair cells.

N = 30 for all observations except for the apical turn (n = 14).

*Student's t-test for SV thickness, internal diameter of vessels in SV, and vessel wall to radius ratio in SV. Wilcoxon rank-sum test for other items.

The density of SG neurons was significantly lower in the DIO group (9.8±2.5/50 µm^2^) than in the control group (12.0±3.6/50 µm^2^) at the basal turn of the cochlea (Wilcoxon rank-sum test, p = 0.0092), but not at the middle turn or the apex.

The density of fibrocytes in the lower part of the SL was lower of borderline significance in the DIO group (6.5±2.6/50 µm^2^) than in the control group (8.6±3.2/50 µm^2^) at the basal turn of the cochlea (Wilcoxon rank-sum test, p = 0.0515), but not at the middle turn or the apex.

In the Organ of Corti, IHC and OHCs degenerated mildly at the apex and moderately at the middle and basal turns, but the extent of OHC loss was similar in both groups (Table 3).

**Figure 1:**
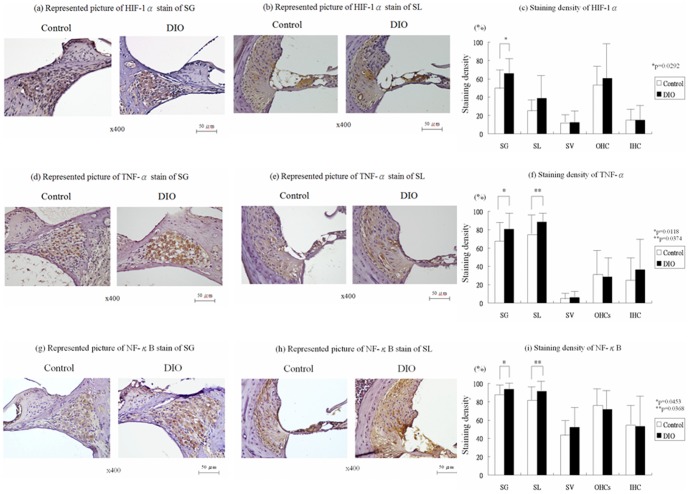
Immunohistochemical studies of cochlea. Representative radial section of hypoxia-induced factor (HIF) 1-α staining in the cochlear basal turn (a,b). The intensity of HIF-1α stain was significantly greater in the DIO group than in the control group in the SG and SL (c). Representative radial section of TNF-α stain in the cochlear basal turn (d,e). The intensity of tumor necrosis factor (TNF)-α stain was significantly tenser in the DIO group than in the control group in SG and SL (f). Representative radial section of NF-κB stain in the cochlear basal turn (g,h). The intensity of nuclear factor kappaB (NF-κB) stain was significantly greater in the DIO group than in the control group in SG and SL (i).

Figures 1a and b show a representative radial section of HIF-1α staining in the basal turn of the cochlea. HIF-1α was expressed moderately in SG and OHCs and mildly in SL, SV, and IHC in both groups. Compared to the control group, the DIO group showed significantly increased HIF-1α staining in the SG (65.8±16.7% vs. 50.0±19.7%; Wilcoxon rank-sum test, p = 0.0292) (Figure 1c).

Figures 1d and e show a representative radial section of TNF-α staining in the basal turn of the cochlea. TNF-α was expressed moderately in the SG and SL and mildly in the SV and the Organ of Corti of the cochlea in both groups. Compared to the control group, the DIO group showed significantly increased TNF-α staining in the SG (80.2±17.9% vs. 67.5±21.0%; Wilcoxon rank-sum test, p = 0.0118) (Figure 1f) and the SL (88.6±8.9% vs. 74.6±22.6%; Wilcoxon rank-sum test, p = 0.0374) (Figure 1f).

Figures 1g and h show a representative radial section of NF-κB staining in the basal turn of the cochlea. NF-κB expression was high in the SG, SL, and OHCs, and moderate in the SV and IHC in both groups. Compared to the control group, the DIO group showed significantly increased NF-κB staining in the SG (93.7±6.7% vs. 87.7±12.7%; Wilcoxon rank-sum test, p = 0.0453) (Figure 1i) and the SL (91.5±9.5% vs.81.5±16.4%; Wilcoxon rank-sum test, p = 0.0368) (Figure 1i).

**Figure 2:**
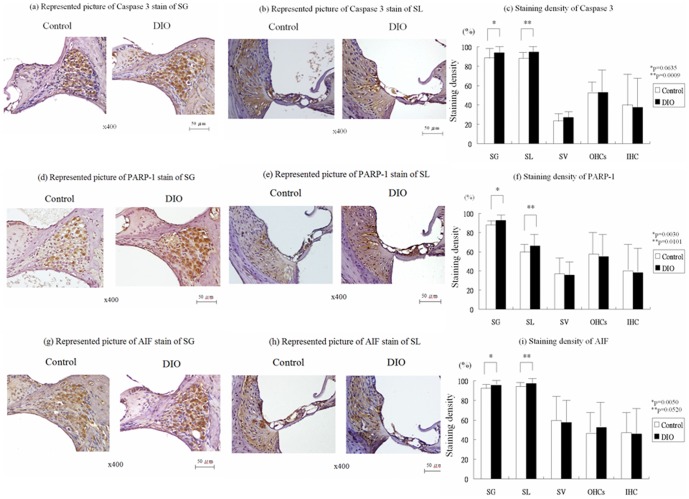
Immunohistochemical studies of cochlea. Representative radial section of caspase 3 staining in the cochlear basal turn (a,b). The intensity of caspase 3 stain was significantly tenser in the DIO group than in the control group in SG and SL (c). Representative radial section of poly(ADP-ribose) polymerase-1 (PARP-1) stain in the cochlear basal turn (d,e). The intensity of PARP-1 stain was significantly greater in the DIO group than in the control group in SG and SL (f). Representative radial section of apoptosis inducing factor (AIF) stain in the cochlear basal turn (g,h). The intensity of AIF stain was significantly tenser in the DIO group than in the control group in SG and SL (i).

Figures 2a and b show a representative radial section of caspase 3 staining in the basal turn of the cochlea. Caspase 3 was stained intensely in the SG and SL, mildly in the SV, and moderately in the Organ of Corti in both groups. Compared to the control group, the DIO group showed increased caspase 3 staining in the SG (93.8±6.6 % vs. 88.6±11.1%; Wilcoxon rank-sum test, p = 0.0635) (Figure 2c) and significantly in the SL (94.7±6.5% vs. 88.3±7.2%; Wilcoxon rank-sum test, p = 0.0009) (Figure 2c).

Figures 2d and e show a representative radial section of PARP-1 staining in the basal turn of the cochlea. PARP-1 was stained intensely in the SG, moderately in the SL and the Organ of Corti, and mildly in the SV of the cochlea in both groups. Compared to the control group, the DIO group showed significantly increased PARP-1staining in the SG (92.8±5.9% vs. 87.8±4.6%; Wilcoxon rank-sum test, p = 0.0030) (Figure 2f) and the SL (66.4±10.6% vs. 59.8±9.2%; Wilcoxon rank-sum test, p = 0.0101) (Figure 2f).

Figures 2g and h show a representative radial section of AIF staining in the basal turn of the cochlea. AIF was stained intensely in the SG and SL and moderately in the SV and the Organ of Corti in both groups. Compared to the control group, the DIO group showed significantly increased AIF staining in the SG (95.7±5.2% vs. 92.5±3.0%; Wilcoxon rank-sum test, p = 0.0050) (Figure 2i) and the SL (97.0±3.9% vs. 94.4±4.5%; Wilcoxon rank-sum test, p = 0.0520) (Figure 2i).

## Discussion

This animal study demonstrates that a high fat diet can lead to body weight gain, abdominal fat accumulation, elevated plasma TG, and elevated hearing thresholds (especially at high frequencies) in CD/1 mice. The most notable histological findings resulting from a high fat diet were narrowed blood vessels in the SV, increased inflammatory responses, and increased cell loss in the SG and SL in the basal turn of the cochlea. Furthermore, a high fat diet also resulted in cell death via activation of both caspase-dependent and -independent apoptosis signaling pathways.

Previous studies have shown that genetic susceptibility and environmental factors including noise, ototoxic medication, chemical exposure, tobacco, hormone replacement therapy, diabetes mellitus (DM), hypertension (HTN), dyslipidemia, and socioeconomic status were associated with peripheral hearing dysfunction in humans [16], [17]. Recently, BMI [6] and WC [7] were reported to be novel independent risk factors for hearing impairment in humans. The concentration of plasma adiponectin, one of the anti-inflammatory adipocytokines, was also shown to be negatively associated with hearing threshold in humans [18].

Lee et al. [19] reported that *ob/ob* mice, a model for type 2 diabetes and obesity [20], exhibited early sensorineural hearing loss. Vasilyeva et al. [21] reported that the hearing functions of CBA/CaJ mice with type 2 DM (HFD-induced) degenerated more rapidly than those of CBA/CaJ mice with type 1 DM (streptozotocin-induced), even though blood glucose levels were higher in type 1 DM mice. We therefore propose that, in addition to glucotoxicity, obesity itself and its related lipotoxicity might be responsible for hearing impairment.

Previous animal studies and our current study have all shown that a high fat diet can lead to hearing impairment through cochlear microangiopathy, neuropathy, and/or damage to the Organ of Corti [22], [23]. Loss of SG cells and degeneration of outer hair cells were observed in the middle and basal turns of the cochlea in *ob/ob* mice [19]. The pathological findings of the current study were similar to those observed in *ob/ob* mice in Lee's study [19]. Their study found that obesity in *ob/ob* mice was caused by leptin deficiency and that hyperglycemia might confound the process of hearing degeneration. In contrast, diet-induced obesity in our study often presented with hyperleptinemia [24], which reflects the actual state that commonly occurs in human obesity [25]. Secondly, plasma TG was only slightly higher in the DIO group than in the control group in our study. Thus, our results were less confounded by plasma glucose concentrations.

Apoptosis is regulated by at least two important signaling pathways: caspase-dependent and caspase-independent. Caspase 3 is the effector in the caspase-dependent apoptosis signaling pathway, whereas PARP-1, AIF, and endonuclease G (EndoG) play key roles in the caspase-independent apoptosis signaling pathway. Oxidative stress or reactive oxidative species can cause DNA damage and activate PARP-1, which enters mitochondria and activates AIF. During apoptosis, AIF is released from the mitochondrial intermembrane space to the cytosol and nucleus, independent of Bid for mitochondrial release, activating EndoG and finally leading to cell death [26]. AIF plays a key role in neurodegenerative diseases [27] and several other disease processes [28], [29]. For example, AIF and mitochondrial fusion might play important roles in cerebellar Purkinje cell loss in Harlequin mice [30], seizure-induced neuronal death [31], apoptosis of retinal cells in RCS rats [28], and apoptosis of semi-tendinosus muscle cells in older humans [29].

In fact, both caspase-dependent and -independent apoptotic mitochondrial pathways may be involved in many diseases, including ischemia/reperfusion-related brain damage [26], [32], death of dopaminergic neurons in the substantia nigra of Parkinson's disease patients [33], and retinitis pigmentosa [34]. Lipotoxicity could also lead to cell apoptosis via caspase-dependent and -independent signaling pathways in pancreatic beta-cell lines [11].

Riva et al. [13] reported that the mechanisms of cochlear degeneration in CD/1 mice involve hypoxia, increased HIF-1α and oxidative stress, increased expression of TNF-α, and increased cell death in the cochlea. The Schwann cells of the SG were observed to be more vulnerable to free radical damage than were the neurons, and they degenerated more rapidly in CD/1 mice [13]. The signaling pathway responsible for cell apoptosis in the cochlea of CD/1 mice involved the activation of caspase-3 and Bax-mediated apoptosis via p53 protein accumulation [13]. Here, our results show that obesity increased hypoxic (HIF-1α) and inflammatory responses (TNF-α and NF-κB) in the cochlea of CD/1 mice, especially in the SG and SL. Diet-induced obesity also increased apoptosis of inner ear cells via caspase-independent signaling pathways in CD/1 mice.

It may be argued that diet-induced obesity exacerbates hearing dysfunction indirectly via its co-morbidities [1]–[5]. As mentioned in the introduction, we chose CD/1 mice as the animal model for this study to allow earlier detection of hearing degeneration and to avoid potential interference from other metabolic abnormalities [12]. However, we still cannot entirely exclude these possibilities. Although the fasting plasma glucose and HDL were similar in both groups, lipotoxicity might occur earlier than glucotoxicity in the pathogenesis of diabetic mellitus, as reported in previous studies [35]. Obesity is independently associated with increased risk of CKD in non-diabetic and non-hypertensive adults [36], [37]. Central obesity or waist circumference has been reported to independently predict both CKD and end-stage renal diseases [38]. Obesity is also reported to interact with non–insulin-dependent diabetes mellitus in affecting cochlear dysfunction [21], [39]. Therefore, we believe that obesity itself might also cause hearing impairment early and directly via lipotoxicity.

There are some limitations in this study. While the mice in the control group were fed a standard diet, some of them became overweight at the end of the study, possibly confounding the results. A lean control group is needed to determine the extent to which auditory degeneration is truly influenced by weight gain. Another limitation of the study is that the control and DIO groups were not consuming the same diet. It is difficult to determine whether the observed differences in ABR and histopathology are due to weight gain or the intake of dietary fat.

In conclusion, diet-induced obesity could exacerbate the hearing impairment observed in CD/1 mice. The most notable histological findings were narrowed blood vessels in the SV, increased inflammatory responses, and increased cell loss in the SG and SL (especially in the basal turn of the cochlea) in CD/1 mice. A high fat diet might increase the death of inner ear cells by activating both caspase-dependent and caspase-independent apoptosis signaling pathways.

## Materials and Methods

### Animals

Sixty 4-week-old male CD/1 mice (BioLASCO Taiwan Co., Ltd.) with normal external and middle ear parameters were used in these experiments. The mice had hearing thresholds≤50 decibel sound pressure level (dB SPL) as determined by sound stimulation using 8 kHz tone bursts.

The mice were randomly divided into two groups at the age of 4 weeks. For 16 weeks, the “DIO” group was fed the AIN-93G modified diet to provide 60% of the calories from fat (DIO Series Diets: D12492, Research Diets Company, NJ, U.S.A.) and the “control” group was fed the AIN-93G standard diet, which provides 13.43 % of the calories from fat (Fwusow Industry Co., Ltd, Taiwan).

Animals were housed with 5 mice per cage in a temperature-controlled room on a constant 12 hr light/dark cycle. Food and tap water were available freely throughout the experiments. The Institutional Animal Care and Use Committee of Buddhist Dalin Tzu Chi General hospital approved the protocol used in this study.

### Body weight, plasma biochemistry and auditory brainstem responses (ABR)

Body weight (BW), plasma biochemistry, and ABR were measured at the beginning (at 4 weeks old) and end of the study (at 20 weeks old) under general anesthesia by intraperitoneal injection of pentobarbital (35 mg/kg).

ABR was conducted in a double-walled, soundproof booth. Click sounds and tone bursts (8 kHz, 16 kHz, and 32 kHz) were delivered sequentially via earphones (Telephonics Corp., Farmingdale, NY). Hearing thresholds of these four stimuli were determined by the presence of ABR waves (Intelligent Hearing Systems, Miami, FL).

After an overnight fast, blood samples (about 1.0 ml) were obtained by medial canthus puncture, and the blood was placed in heparin-coated capillary tubes. The plasma was retrieved immediately after centrifugation and was sent for biochemical analysis. Plasma glucose, triglyceride (TG), and high density lipoprotein (HDL) concentrations were measured by hexokinase-glucose-6-phosphate dehydrogenase method, enzymatic method, and homogeneous method using SIEMENS Dimension RxL Max. Chemistry Ananlyzer (SIEMENS), respectively.

### Omental fat weight and histology of the cochlea

At the end of the study, omental fat was excised for weight measurements under general anesthesia with intraperitoneal injection of pentobarbital (35 mg/kg).

Animals were intracardially perfused with 2.5% glutaraldehyde and 4% paraformaldehyde in phosphate buffered saline (PBS: NaCl, 150 mM; KH2PO4, 2 mM; Na2HPO4 2H2O, 8 mM, pH 7.4) before being euthanized. The temporal bones were removed, and the cochlea was perfused with the above fixative through the oval and round windows to an outlet in the apex. After 24-hr post-fixation in the same fixative at 4°C, the temporal bones were decalcified in 10% EDTA solution, 4% paraformaldehyde, and 2.5% glutaraldehyde in PBS (pH 7.4) for 1 week at room temperature. The temporal bones were then rinsed in PBS, dehydrated through a graded series of alcohol and xylene, and embedded in paraffin. Embedded cochleae were sectioned at 5 µm thickness and the cochlear structures were observed at the mid-modiolar level using a light microscope.

#### Hematoxylin and Eosin (H & E) staining

The density of SG neurons and fibrocytes in the lower part of the SL within a central region of 50×50 µm^2^ were measured under ×400 magnification. The thickness of the middle part of the stria vascularis (SV), the mean internal diameter, and the vessel wall to radius ratio of the SV vessels in the SV were calculated under ×400 magnification. The morphology and numbers of outer and inner hair cells in the Organ of Corti were observed under ×400 magnification.

The above features were observed in two adjacent cochlear sections at the same mid-modiolar level and averaged for each mouse.

#### Immunohistochemical staining

The sections were heat-treated at 95°C in 10 mM sodium citrate buffer for 20 min, incubated in 3% H_2_O_2_ for 10 min at room temperature, and blocked in 5% skim milk to prevent non-specific labeling. The specimens were then incubated overnight at 4°C with one of the following primary antibodies: mouse monoclonal anti-hypoxia-induced factor (HIF) 1-α (1∶250 dilution) (Abcam plc, Cambridge, U.K.), rabbit polyclonal anti-tumor necrosis factor (TNF)-α (1∶500 dilution) (Abbiotec, LLC, San Diego, U.S.A.), rabbit polyclonal anti-nuclear factor kappaB (NF-κB) p65 (1∶1000 dilution) (Abcam plc, Cambridge, U.K.), rabbit anti-caspase 3 (1∶500 dilution) (R&D systems, Inc., U.S.A.), rabbit monoclonal anti-poly(ADP-ribose) polymerase-1 (PARP-1) (1∶500 dilution) (Abcam plc, Cambridge, U.K.), or rabbit anti-apoptosis inducing factor (AIF) (1∶1000 dilution) (R&D systems, Inc., U.S.A.). Slides were washed in PBS three times, exposed to a secondary antibody for 1 h at room temperature, and stained using the DAB staining kit (R&D systems, Inc., U.S.A.) according to the manufacturer's protocol. Slides were dehydrated, mounted, and observed through a light microscope. The images were acquired using a CCD camera (Dage-MTI Inc, Michigan City, Ind) connected to a personal computer and analyzed using image analysis software (Image-Pro Plus, version 6.0; Media Cybernetics, Silver Springs, MD).

The structures in question were observed in two adjacent cochlear sections at the same mid-modiolar level and averaged for each mouse. Stained regions were quantified as the percentage of the entire target area [15].

### Statistical analysis

The data were presented as means±standard deviation (SD) unless indicated otherwise. BW, biochemistry, omental fat weight, ABR thresholds, and histological findings were compared between the two study groups using the Student's t-test if the data show a normal distribution or Wilcoxon rank-sum test if the data did not show a normal distribution. All analyses were performed using STATA 10.0 software (Stata Corp, L.P., College Station, TX). P values<0.05 were considered statistically significant.
